# A Real-Time Magnetic Adhesion Force Estimation Method for Wall-Climbing Robots Equipped with Halbach Permanent Magnet Arrays

**DOI:** 10.3390/s26092678

**Published:** 2026-04-25

**Authors:** Jiabin Cao, Lin Zhang, Yiyang Zhao, Ming Chen

**Affiliations:** 1 School of Mechanical Engineering, Shanghai Jiao Tong University, Shanghai 200240, China; caojiabin@sjtu.edu.cn (J.C.); 804311109@sjtu.edu.cn (Y.Z.); 2 School of Robot Engineering, Yangtze Normal University, Chongqing 408100, China; lin.zhang_2014@hotmail.com

**Keywords:** magnetic adhesion force, Halbach magnet array, magnetostatic method of images, wall-climbing robot

## Abstract

This paper presents a real-time magnetic adhesion force estimation framework for wall-climbing robots equipped with Halbach permanent magnet arrays (PMAs) and air-gap–adjustable mechanisms. Accurately computing the magnetic adhesion force between a PMA and a large ferromagnetic surface is challenging due to the nonlinear magnetization behavior of soft magnetic materials and the strongly coupled, highly nonuniform magnetic fields generated by Halbach arrays. Conventional analytical models fail to capture these effects, while finite element methods (FEM) incur prohibitive computational cost for real-time applications. To address this, we propose an analytical magnetic-force estimation model based on the magnetostatic MoI (Method of Images), which replaces the unknown magnetization inside the steel plate with an equivalent image magnet distribution that satisfies boundary conditions at the air–steel interface. The method avoids solving complex magnetization in soft magnetic media and enables a unified force computation for arbitrarily oriented magnet elements. Additionally, complex Halbach PMA geometries are approximated through cuboid-element segmentation into cuboid magnet array, allowing efficient force evaluation. Comparative studies demonstrate that the proposed method achieves accuracy comparable to FEM while reducing computation time by several orders of magnitude. Experimental validation using a linear Halbach array and a large steel plate proved that the framework can reliably estimate magnetic adhesion force across varying air-gap distances, meeting the real-time requirements of air-gap–adjustable wall-climbing robots.

## 1. Introduction

Wall-climbing robots have attracted increasing attention due to their capability to operate on vertical and inverted surfaces in industrial inspection, maintenance, and hazardous environments. According to recent reviews on the design and technical development of wall-climbing robots [1,2,3], various adhesion mechanisms have been developed to enable stable attachment to surfaces, including vacuum suction, aerodynamic adhesion, bio-inspired dry adhesion, electromagnetic adhesion, and permanent magnetic adhesion. Among these mechanisms, permanent magnetic adhesion has become one of the most widely adopted solutions for wall-climbing robots operating on ferromagnetic surfaces, due to its inherent advantages of zero energy consumption, strong and stable adhesion force, and high operational safety. By utilizing permanent magnet arrays (PMAs) to generate strong adhesion at small air gaps, these robots can reliably carry inspection, maintenance, and manipulation equipment in challenging environments. To ensure operational safety, the magnetic adhesion force is typically designed with a significant safety margin. However, since the friction force is directly proportional to the normal magnetic force, excessive adhesion leads to increased locomotion resistance, reduced maneuverability, and higher energy consumption. This trade-off has driven the development of wall-climbing robots equipped with air-gap–adjustable or tunable adhesion mechanisms, where real-time knowledge of the magnetic adhesion force becomes essential for adaptive control and efficient operation. Beyond industrial robotics, similar challenges in magnetic force modeling also arise in magnetically actuated robotic systems in the biomedical domain. For instance, magnetic anchored and guided endoscopes [4] rely on external magnetic fields to generate controllable interaction forces for minimally invasive procedures. Recent studies have further demonstrated the integration of advanced sensing–modeling–control strategies in robotic endoscopy, including image-based visual servoing methods for automatic view regulation [5] and neural-network-based control approaches for magnetically actuated capsule endoscopic robots [6], which address target tracking, motion constraints, and robustness against disturbances. In such systems, accurate and computationally efficient magnetic force estimation is essential for enabling real-time feedback and closed-loop control. Therefore, the magnetic force computation strategy investigated in this work is not only beneficial for wall-climbing robots but also provides a generalizable solution that can support sensing–modeling–control integration in a broader class of magnetically actuated robotic systems.

Accurate estimation of magnetic adhesion force remains a challenging problem due to the nonlinear magnetization behavior of ferromagnetic materials and the highly nonuniform magnetic fields generated by complex PMA configurations. Existing approaches can be broadly classified into measurement-based and modeling-based methods. Measurement-based approaches rely on force sensors or indirect sensing strategies, which often suffer from limited range, additional system complexity, and high cost. In contrast, modeling-based approaches aim to predict the force through physical formulations and mainly include analytical models [7] and numerical solutions [8].

As one of the most widely adopted numerical methods, finite element methods (FEM) [9,10] offer high accuracy and strong generality by solving Maxwell’s equations numerically and have been commonly used for magnetic force analysis in wall-climbing robotics. For example, Zhu et al. [11] analyzed magnetic field distributions and force fluctuations using Maxwell simulations. Despite their accuracy, FEM-based methods rely on dense meshing and iterative solvers, resulting in substantial computational costs that limit their use in real-time applications. To improve computational efficiency, various advanced numerical approaches have been proposed for magnetic field and force calculations, including the boundary element method [12] and other hybrid methods [13]. Nevertheless, these methods still depend on discretization and iterative procedures and thus remain computationally demanding for real-time force estimation tasks.

In the field of magnetic field computation, common analytical methods include the magnetic equivalent circuit (MEC) method [14], tooth-profile method [15], surface charge/current models [16,17], and the magnetostatic MoI [18,19]. The MEC method, which draws an analogy between magnetic circuits and electrical circuits, offers fast computation and can account for material saturation, but it lacks detailed field accuracy. As an attempt to improve the arruracy, Wang et al. [20] combined MEC models with FEM validation to study adhesion forces under varying robot configurations. The tooth-profile method improves precision by dividing regions of different permeability into a network, yet it requires complex modeling and recomputation for each rotor position. Surface charge/current models and the magnetostatic MoI can handle 2D or 3D configurations and finite-permeability materials, but their applicability is limited for high-permeability media or complex boundaries. Janssen et al. [19] proposed a closed-form analytical method combining the surface charge model and the magnetostatic MoI, which can quickly predict the force between permanent magnets and soft magnetic components and is helpful for more efficient design of magnetic levitation systems. However, their work mainly focused on common cuboid magnets and failed to address the extension to complex-shaped magnets. Ortner et al. [21] introduced Magpylib, a Python-based analytical library for magnetic field computation, which is built upon several established closed-form solutions for permanent magnets and current-carrying elements. In addition to cuboid magnets, the library also supports cylindrical magnets, triangular surface charges, and current loops, substantially broadening its applicability. Liang et al. [22] proposed a matrix-based framework based on the surface charge model for efficient magnetic field and force evaluation, which introduced cuboid segmentation and approximation to facilitate the calculation for arc-shaped magnets and other complex-shaped magnets. However, the magnetic force in the framework is derived indirectly through the integral of the magnetic flux density, which still requires further refinement to meet the demands of real-time applications. In the context of direct force-based magnetic field analysis, Allag et al. [23] presented the analytical expression for the interaction force between two parallelepipedic magnets based on 3D analytical calculation theory. Because the closed-form formulas directly yield the magnetic forces, the computational efficiency is significantly higher compared with indirect methods. However, their approach remains limited to cuboid PMs or PMAs and does not extend to more complex scenarios involving non-standard magnet geometries or ferromagnetic components. Overall, these methods exhibit inherent trade-offs between accuracy and computational efficiency, making them unsuitable for real-time estimation of adhesion forces in wall-climbing robots.

**Figure 1:**
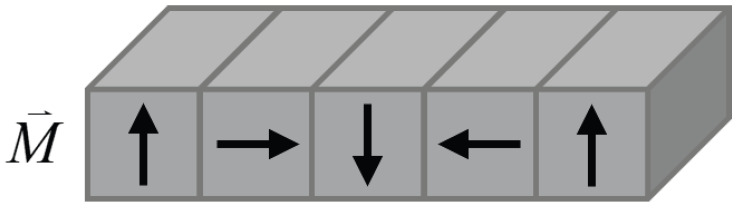
Four-piece linear Halbach PMA used in the adhesion module. Black arrows indicate the magnetization directions.

**Figure 2:**
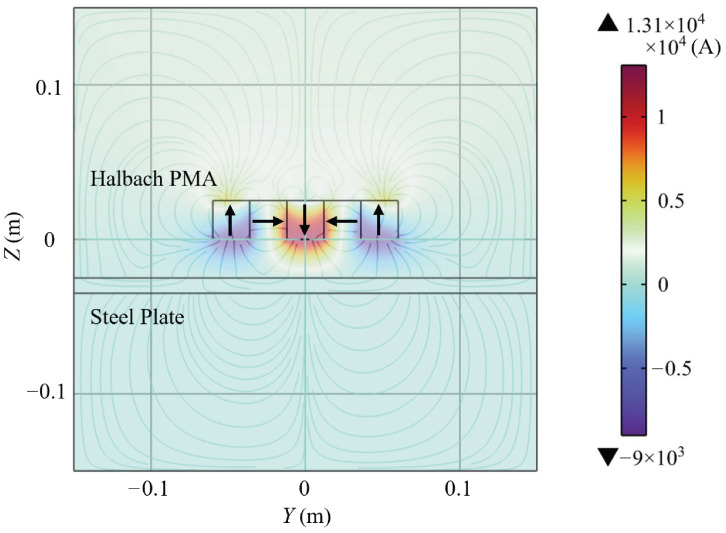
Scalarpotential distribution and magnetic field lines of a linear Halbach PMA near a steel plate simulated in COMSOL Multiphysics 6.1.

Among various PMA configurations, Halbach arrays have attracted considerable attention due to their flux-focusing capability and asymmetric magnetic field distribution. By arranging magnets with spatially varying magnetization directions, Halbach PMAs concentrate magnetic flux on the working surface while suppressing it on the opposite side, thereby enhancing adhesion performance, as illustrated in Figure 1 and Figure 2. However, the resulting strongly coupled and highly nonuniform magnetic field significantly increases modeling complexity. Existing studies have investigated Halbach arrays using both analytical and numerical approaches mentioned above, as well as many hybrid approaches. Mihalache et al. [24] reconstructed magnetization distributions using hybrid methods, while Ye et al. [25] developed analytical models for Halbach guideways. Makridis et al. [26] conducted FEM-based studies on 3D-printed Halbach structures, and Jiao et al. [27] proposed MEC models for wall-climbing robots. Although these works provide valuable insights, a unified framework that can efficiently handle complex Halbach geometries and their interaction with ferromagnetic materials for real-time applications is still lacking.

**Figure 3:**
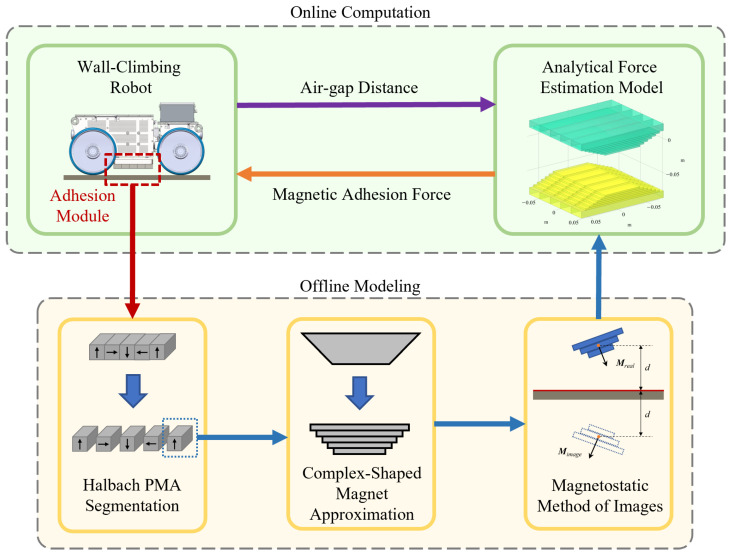
Real-time analytical magnetic adhesion force estimation framework.

To address these gaps, this paper proposes a real-time estimation framework for magnetic adhesion force in wall-climbing robots is proposed by synergistically integrating the magnetostatic MoI, 3D analytical formulations, and segmentation-based approximation strategies. The overall structure of the framework is illustrated in Figure 3. The main contributions of this work are summarized as follows:

- A unified analytical framework is developed that combines the advantages of the magnetostatic method of images and 3D analytical force computation. By replacing explicit magnetization solving in soft magnetic materials with image-based equivalents, the proposed method avoids computationally intensive procedures and enables fast evaluation of magnetic adhesion forces.
- A segmentation and approximation strategy based on cuboid elements is incorporated into the analytical formulation, allowing the framework to extend beyond simple geometries and effectively handle complex-shaped magnets. This includes configurations such as Halbach arrays and arbitrarily shaped permanent magnets.
- The proposed framework is capable of modeling interactions not only between permanent magnets but also with ferromagnetic surfaces, thereby covering more realistic and challenging application scenarios in wall-climbing robots with adjustable air gaps.
- Comparative studies with existing open-source analytical tools and finite element method (FEM) simulations demonstrate that the proposed approach maintains comparable accuracy while significantly improving computational efficiency, making it suitable for real-time applications.

The remainder of this paper is structured as follows. Section 2 presents the methodology of the proposed framework as well as the simulation and experimental setup. Section 3 describes and compares the force estimation results. Finally, Section 4 summarizes the main conclusions and outlines directions for future research.

## 2. Materials and Methods

This section mainly presents a real-time magnetic adhesion force estimation framework for wall-climbing robots. When climbing on plane surfaces, the magnetic adhesion force controlled by the air-gap-adjustable mechanism can be estimated fast and precisely through the framework.

### 2.1. Problem Statement

**Figure 4:**
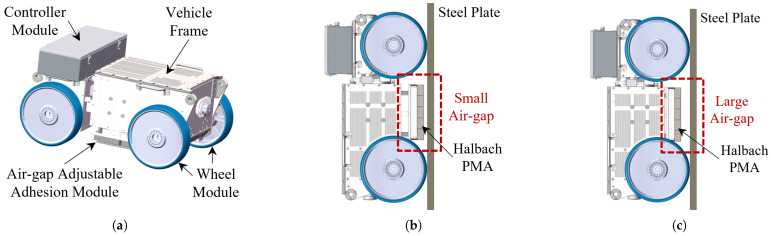
Four-wheeled wall-climbing robot equipped with Halbach PMA and air-gap adjustable mechanism. (**a**) Main modules of the robot system. (**b**) Adhesion with a small air gap. (**c**) Adhesion with a large air gap.

**Figure 5:**
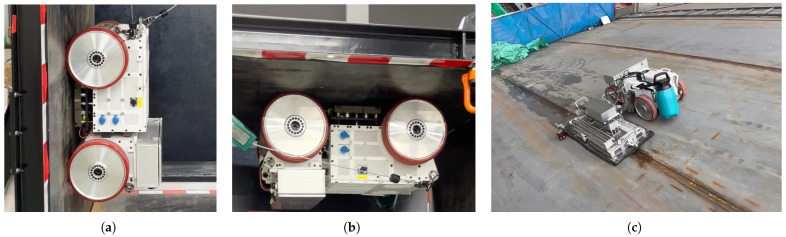
Several scenes for testing the wall-climbing robot. (**a**) Robot climbing on vertical steel surface. (**b**) Robot moving on the steel ceiling. (**c**) Robot carrying cleaning equipment to perform cleaning operations on an inclined steel surface.

As Figure 4a illustrates, the wall-climbing robot studied here mainly consists of vehicle frame, controller module, adhesion module, and four wheel modules. The air-gap distance between the wall surface and the adhesion module is designed to be adjusted by a set of hydraulic linear actuators. Smaller air gaps, as shown in Figure 4b, will result in more stable adhesion, facilitating the stability of the operation when the vehicle is stationary, while larger air gaps, as depicted in Figure 4c, will reduce the friction between the wheels and the wall and will benefit the rapid movement of the robot. The distance could be computed through the stroke-length feedback of the actuator after calibration, which will be used as the input of the magnetic force estimation framework. Figure 5 presents several functional tests of the wall-climbing robot. Equipped with the adhesion module, the robot is capable of operating on flat vertical and inclined surfaces, as well as on ceilings. As illustrated in the representative cleaning scenario in Figure 5c, the application of the MoI model for magnetic force estimation is well justified, since the working steel surface is sufficiently large and planar to be approximated as an ideal infinite boundary.

### 2.2. Segmentation and Approximation of Halbach PMAs

A general cuboid segmentation strategy for complex-shaped magnets is introduced first. Consider a magnet with a constant thickness along the *y* direction and an arbitrary convex polygonal cross-section in the *x*–*z* plane. The polygon is defined by an ordered vertex set:

(1) $$P={\left\{\left(x_{k},z_{k}\right)\right\}}_{k=1}^{n}$$

The objective is to approximate the magnet by a set of cuboid slices for magnetic force evaluation while ensuring a conservative estimation:

(2) $$F_{\mathrm{est}}\leq F_{\mathrm{true}}$$

This conservative formulation is particularly important in magnetic adhesion applications for wall-climbing robots, where a lower-bound estimation of the adhesion force introduces additional safety margin in force control, thereby mitigating the risk of detachment and enhancing system robustness. Meanwhile, this slicing strategy is adopted to ensure compatibility with the subsequent magnetic force computation, where the analytical formulation is derived for interactions between cuboid elements with parallel or orthogonal spatial relationships. By decomposing the magnet into horizontally aligned cuboid slices, the geometric configuration of each element pair satisfies the applicability conditions of the analytical model, enabling efficient and consistent force integration. The cross-section is divided into $N_{z}$ horizontal slices along the *z*-direction:

(3) $$z_{i}=z_{\min}+\left(i-\frac{1}{2}\right)\Delta z,\;\Delta z=\frac{z_{\max}-z_{\min}}{N_{z}}$$

where each slice spans:

(4) $$\left[z_{i}-\frac{\Delta z}{2},\;z_{i}+\frac{\Delta z}{2}\right]$$

For a given horizontal line $z=z_{0}$, intersections with polygon edges are computed via linear interpolation. For an edge connecting $\left(x_{1},z_{1}\right)$ and $\left(x_{2},z_{2}\right)$, the intersection point is:

(5) $$x\left(z_{0}\right)=x_{1}+\frac{z_{0}-z_{1}}{z_{2}-z_{1}}\left(x_{2}-x_{1}\right)$$

For convex polygons, the intersection defines a single interval:

(6) $$\left[x_{L}\left(z_{0}\right),\;x_{R}\left(z_{0}\right)\right]$$

To ensure strict geometric containment, the segment within each slice is constructed using the intersection points at the top and bottom boundaries, including $x_{L}^{\mathrm{top}}=x_{L}\left(z_{i}+\frac{\Delta z}{2}\right)$, $x_{R}^{\mathrm{top}}=x_{L}\left(z_{i}+\frac{\Delta z}{2}\right)$, $x_{L}^{\mathrm{bot}}=x_{L}\left(z_{i}-\frac{\Delta z}{2}\right)$, $x_{R}^{\mathrm{bot}}=x_{L}\left(z_{i}-\frac{\Delta z}{2}\right)$. The conservative interval is defined as:

(7) $$\left[x_{L},x_{R}\right]=\left[\max\left(x_{L}^{\mathrm{top}},x_{L}^{\mathrm{bot}}\right),\min\left(x_{R}^{\mathrm{top}},x_{R}^{\mathrm{bot}}\right)\right]$$

The line segment is defined by its center $\left(x_{i},z_{i}\right)$ and width $l_{i}$:

(8) $$x_{i}=\frac{x_{L}+x_{R}}{2},\;l_{i}=x_{R}-x_{L}$$

For convex polygons, the constructed interval satisfies:

(9) $$\left[x_{L},x_{R}\right]\subseteq \left[x_{L}\left(z\right),x_{R}\left(z\right)\right],\;\forall z\in \left[z_{i}-\frac{\Delta z}{2},\;z_{i}+\frac{\Delta z}{2}\right]$$

Thus, the segmented region is strictly contained within the original geometry, which guarantees a conservative estimation. Near the top or bottom boundaries, the interval may degenerate (i.e., $x_{R}\leq x_{L}$). In such cases, a substitution of the centerline-based approximation is adopted:

(10) $$\left[x_{L}^{\mathrm{mid}},x_{R}^{\mathrm{mid}}\right]=\left[x_{L}\left(z_{i}\right),\;x_{R}\left(z_{i}\right)\right]$$

The segment is then defined as:

(11) $$x_{i}=\frac{x_{L}^{\mathrm{mid}}+x_{R}^{\mathrm{mid}}}{2},\;l_{i}=x_{R}^{\mathrm{mid}}-x_{L}^{\mathrm{mid}}$$

**Figure 6:**
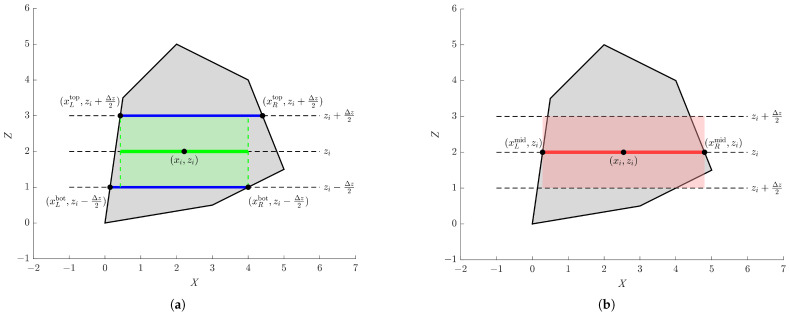
Illustration of 2D slicing and approximation strategy. The gray region denotes an arbitrary polygonal cross-section. The black dashed lines indicate the lower boundary, centerline, and upper boundary of the slice. (**a**) The boundary-based approximation. Blue line segments represent the intervals at the slice boundaries, while the green line segment on the centerline corresponds to the interval intersection. The shaded green region denotes the resulting cuboid slice. (**b**) The centerline-based approximation. Red line segment represents the interval at the slice centerline, and the resulting cuboid slice is denoted with shaded red.

**Figure 7:**
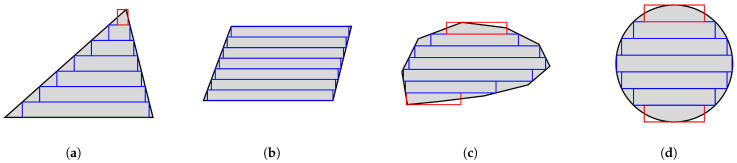
Slicing examples of different cross-section patterns, including (**a**) triangle, (**b**) trapezoid, (**c**) arbitrary convex polygon and (**d**) circle, using the 2D conservative slicing strategy. Blue boxes represent boundary-based approximation, while red boxes represent centerline-based approximation.

Figure 6 illustrates the boundary-based and centerline-based slicing and approximation strategy with a single slice on a random convex polygon, and Figure 7 presents several examples of slicing different cross-section patterns. As can be seen, the proposed strategy ensures strict geometric containment through interval intersection while maintaining numerical robustness via centerline-based correction. It is applicable to arbitrary convex cross-sections and provides a conservative approximation suitable for safe magnetic force estimation. Moreover, as illustrated in Figure 7d, the proposed method can be extended to curved profiles by discretizing them into polygons of numerous short line segments, making them compatible with the polygon slicing approach.

**Figure 8:**
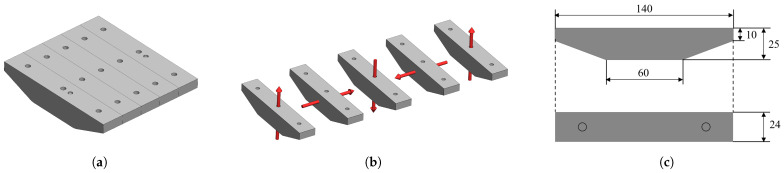
(**a**) CAD model of a linear Halbach PMA composed of five complex-shaped magnets. (**b**) Magnetization permutation of the magnets indicated with red arrows. (**c**) Geometry of the complex-shaped magnet (all dimensions in mm).

The PMs studied in our simulation are arranged in four-piece linear Halbach array form, which indicates perpendicular magnetization directions for adjacent PMs, as illustrated in Figure 8a,b, to enhance the magnetic flux density on one side of the magnetic adhesion module and reduce it on the other side. The dimensions of a single complex-shaped PM are presented in Figure 8c.

**Figure 9:**
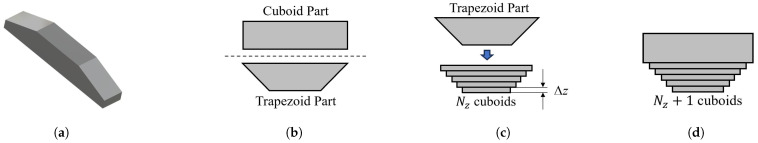
Slicing and approximation of a complex-shaped magnet. (**a**) Model of the simplified magnet. (**b**) Magnet divided into two parts. (**c**) Conservative slicing of the trapezoid part. (**d**) Approximation of the original magnet with cuboid array.

As shown in Figure 8a, the actual magnets include structural features such as mounting and positioning holes. For computational convenience, these features are neglected, resulting in the simplified model shown in Figure 9a. The resulting geometry can be decomposed into a cuboid part and a trapezoid part, as illustrated in Figure 9b. The trapezoid part is then discretized using the proposed boundary-based conservative approximation. Specifically, the cross-section is partitioned into $N_{z}$ horizontal slices along the height direction. Each slice is approximated by a cuboid constructed from the 2D conservative slicing strategy described above, as shown in Figure 9c. Finally, the complete magnet model is obtained by combining the cuboid part with the $N_{z}$ slices, yielding a conservative cuboid-based equivalent for subsequent magnetic force computation, as shown in Figure 9d.

Then, the total magnetic attraction force is obtained by summing up the magnetic attraction force elements of each part:

(12) $$F=\sum_{i=1}^{N}\Delta F_{i}$$

When a permanent magnet approaches a large steel plate, the plate becomes magnetized and can be equivalently regarded as a nonuniformly magnetized magnet. By discretizing the plate into sufficiently small subregions, each subregion can be approximated as a uniformly magnetized magnetic element. The magnetic attraction between the permanent magnet and each element can then be computed using the analytical force formula for two magnets, and the total force is obtained by summing the contributions of all elements. However, due to the complexity of magnetic induction within the steel, it is extremely difficult to analytically determine the magnetization of every element. Even if such magnetization could be computed, a very fine discretization would be required to maintain accuracy, resulting in prohibitive computational cost and poor efficiency.

To address this problem, we adopted the magnetostatic method of images to replace the interaction between the permanent magnet and the magnetized steel plate with the interaction between the source magnet and its image magnet. In this way, the difficulty of solving the magnetization distribution inside the steel plate in real time is effectively avoided.

### 2.3. Adhesion Force Model Based on Magnetostatic Method of Images

**Figure 10:**
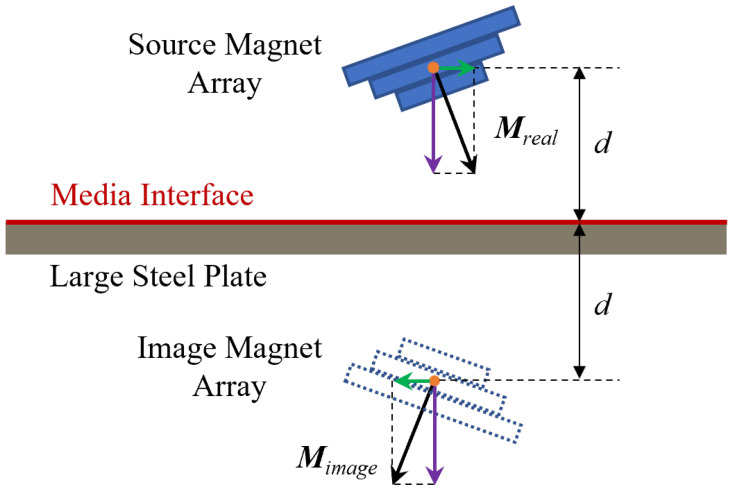
Magnetostatic method of images for PMA.

As illustrated in Figure 10, the magnetic adhesion force between a PMA and a large ferromagnetic plate can be computed using the magnetostatic method of images under several standard assumptions:

1. The steel plate is sufficiently large and thick such that magnetic leakage at the boundaries or magnetic saturation effect is negligible.
2. The plate surface can be regarded as an infinite planar interface, allowing the symmetry conditions required for the image method to hold.

According to classical magnetostatics, the method of images replaces the ferromagnetic medium by constructing an image magnet array located symmetrically with respect to the media interface. The geometry of the image array is fully mirrored about the interface. To satisfy the continuity conditions of the magnetic field at the boundary—namely, that the normal component of the magnetic flux density is continuous while the tangential component vanishes—the magnetization vector of the image array is defined by reversing the tangential components relative to the interface while keeping the normal component unchanged.

Under this construction, the magnetic attraction exerted by the PMA on the steel plate becomes mathematically equivalent to the magnetic force between the PMA and its corresponding image array. Because the image array is generated analytically and does not require solving the magnetization distribution inside the soft magnetic material, the resulting formulation significantly simplifies the computation of magnetic force. Moreover, once the image array is established, the magnetic interaction can be evaluated directly using closed-form expressions or rapidly converging numerical summations, thereby enabling efficient force estimation suitable for real-time applications.

**Figure 11:**
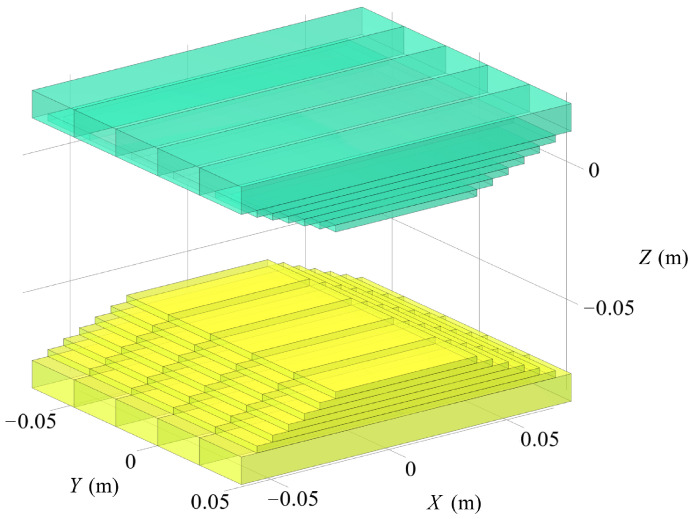
The equivalent source PMA (green) and its image array (yellow), each located 29 mm from the media interface.

The equivalent magnet array obtained from the segmentation and approximation of the complex-shaped magnet described in the previous section, together with the corresponding image array constructed using the magnetostatic MoI, is illustrated in Figure 11. These arrays are used in the subsequent magnetic adhesion force estimation algorithm. The overall procedure of the slicing strategy is presented in Algorithm 1.
**Algorithm 1** Conservative 2D Slicing via Boundary Interval Intersection**Require:** Polygon P, number of slices $N_{z}$**Ensure:** Slice set S1:Compute $z_{\min},z_{\max}$ and Δz2:Initialize S←∅3:**for** i=1 to $N_{z}$ **do**4:      $z_{i}\leftarrow z_{\min}+\left(i-\frac{1}{2}\right)\Delta z$5:      Compute intervals at:$$z_{i}+\frac{\Delta z}{2},\;z_{i}-\frac{\Delta z}{2},\;z_{i}$$6:      Construct conservative interval:$$\left[x_{L},x_{R}\right]=\left[\max\left(x_{L}^{\mathrm{top}},x_{L}^{\mathrm{bot}}\right),\;\min\left(x_{R}^{\mathrm{top}},x_{R}^{\mathrm{bot}}\right)\right]$$7:      **if** $x_{R}\leq x_{L}$ **then**8:           Use centerline interval at $z_{i}$:$$x_{i}=\left(x_{L}^{\mathrm{mid}}+x_{R}^{\mathrm{mid}}\right)/2,\;l_{i}=x_{R}^{\mathrm{mid}}-x_{L}^{\mathrm{mid}}$$9:      **else**$$x_{i}=\left(x_{L}+x_{R}\right)/2,\;l_{i}=x_{R}-x_{L}$$10:     **end if**11:     Add cuboid slice $\left(x_{i},z_{i},l_{i},\Delta z\right)$ to S12:**end for**13:**return** S

After introducing the method of images, the magnetic force expressed in Equation (12) can be reformulated as Equation (13):

(13) $$F=\sum_{i=1}^{N}\sum_{j=1}^{N}\Delta F_{ij}$$

where $\Delta F_{ij}$ denotes the magnetic force between the *i*-th magnet in the source magnet array and the *j*-th magnet in the corresponding image array.

### 2.4. Analytical Model of Magnetic Force Between Magnets

As presented by Allag et al. [23], the interaction between two parallelepipedic magnets can be computed in an analytical form based on the 3D analytical calculation theory.

**Figure 12:**
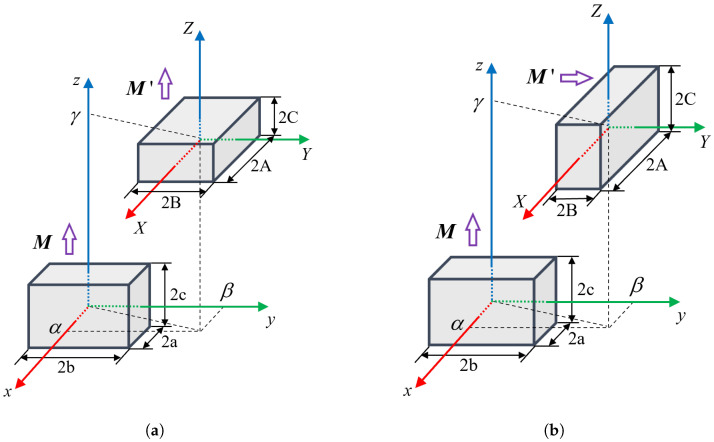
Configuration of two magnets with (**a**) parallel magnetization and (**b**) perpendicular magnetization.

When two permanent magnets have parallel magnetization directions and are arranged as shown in Figure 12a, their interaction force vector F can be computed in the form of Equation (14).

(14) $$\begin{array}{c} F=\frac{∥M∥\cdot ∥M^{\prime }∥}{4\pi \mu_{0}}\sum_{i=0}^{1}\sum_{j=0}^{1}\sum_{k=0}^{1}\sum_{l=0}^{1}\sum_{p=0}^{1}\sum_{q=0}^{1}{\left(-1\right)}^{i+j+k+l+p+q}\cdot \phi \left(U_{ij},V_{kl},W_{pq},r\right) \end{array}$$

$$\begin{array}{c} \phi_{x}\left(U,V,W,r\right)=\frac{\left(V^{2}-W^{2}\right)}{2}\ln\left(r-U\right)+UV\ln\left(r-V\right)+VW\cdot \tan^{-1}\left(\frac{UV}{Wr}\right)+\frac{1}{2}U\cdot r \end{array}$$

$$\begin{array}{c} \phi_{y}\left(U,V,W,r\right)=\frac{\left(U^{2}-W^{2}\right)}{2}\ln\left(r-V\right)+UV\ln\left(r-U\right)+UV\cdot \tan^{-1}\left(\frac{UV}{Wr}\right)+\frac{1}{2}V\cdot r \end{array}$$

$$\begin{array}{c} \phi_{z}\left(U,V,W,r\right)=-UV\ln\left(r-U\right)-VW\ln\left(r-V\right)+UV\cdot \tan^{-1}\left(\frac{UV}{Wr}\right)-W\cdot r \end{array}$$

In contrast, when the two magnets have perpendicular magnetization directions, as illustrated in Figure 12b, the corresponding interaction force follows the formulation of Equation (15).

It can be seen that the overall computational structure of the force remains the same, because in both cases it is obtained from the principle of virtual work by taking the negative gradient of the magnetostatic interaction energy between the two magnets. The difference arises in the specific expression of ϕ(U,V,W,r), since the integral term used in the interaction energy derivation takes different forms under different magnetization orientations.

(15) $$\begin{array}{c} F=\frac{∥M∥\cdot ∥M^{\prime }∥}{4\pi \mu_{0}}\sum_{i=0}^{1}\sum_{j=0}^{1}\sum_{k=0}^{1}\sum_{l=0}^{1}\sum_{p=0}^{1}\sum_{q=0}^{1}{\left(-1\right)}^{i+j+k+l+p+q}\cdot \phi \left(U_{ij},V_{kl},W_{pq},r\right) \end{array}$$

$$\begin{array}{cc} \phi_{x}\left(U,V,W,r\right)= & -VW\ln(r-U)+VU\ln(r+W)+UW\ln(r+V)\\ & -\frac{U^{2}}{2}\tan^{-1}\left(\frac{VW}{Ur}\right)-\frac{V^{2}}{2}\tan^{-1}\left(\frac{UW}{Vr}\right)-\frac{W^{2}}{2}\tan^{-1}\left(\frac{UV}{Wr}\right) \end{array}$$

$$\begin{array}{c} \phi_{y}\left(U,V,W,r\right)=\frac{\left(U^{2}-V^{2}\right)}{2}\ln\left(r+W\right)-UW\ln\left(r-U\right)-UV\cdot \tan^{-1}\left(\frac{UW}{Vr}\right)-\frac{1}{2}W\cdot r \end{array}$$

$$\begin{array}{c} \phi_{z}\left(U,V,W,r\right)=\frac{\left(U^{2}-W^{2}\right)}{2}\ln\left(r+V\right)-UV\ln\left(r-U\right)-UW\cdot \tan^{-1}\left(\frac{UV}{Wr}\right)-\frac{1}{2}V\cdot r \end{array}$$

In the above equations, the secondary variables are:

$$U_{ij}=\alpha +{\left(-1\right)}^{j}A-{\left(-1\right)}^{i}a$$

$$V_{kl}=\beta +{\left(-1\right)}^{l}B-{\left(-1\right)}^{k}b$$

$$W_{pq}=\gamma +{\left(-1\right)}^{q}C-{\left(-1\right)}^{p}c$$

$$r=\sqrt{U_{ij}^{2}+V_{kl}^{2}+W_{pq}^{2}}$$

With the equations above, the magnetic force component between single magnets in the source and image array can be calculated. Finally, they are summed up according to Equation (13) to produce the total magnetic force.

### 2.5. Simulation and Experimental Setup

The code for the proposed magnetic force estimation framework was programmed in MATLAB 2023b, which is also the deployment environment for MagTetris. And FEM-based force calculations were implemented in the COMSOL Multiphysics platform for comparison. All computations and simulations were conducted with the following desktop computer configuration:

- CPU: Intel(R) Core(TM) i5-12400F 2.50 GHz.
- RAM: 16.0 GB.

As the number of cuboid slices $N_{z}+1$ described in Section 2.2 affects both accuracy and computation cost of the framework, an accuracy–efficiency trade-off analysis was implemented to determine a preferred value. The air gap is fixed to 2 mm, and the remanent magnetization of all PMs is set to 1.45 T. The number of cuboid slices ranges form 2 to 21. The estimated magnetic force and the average computation time changing with the number of slices were recorded. As the slicing becomes finer, the estimated magnetic adhesion force gradually increases and asymptotically approaches a constant value. Using the curve fitting toolbox in MATLAB 2023b, the relationship can be fitted with a four-parameter logistic (logistic4) function, as expressed in Equation (16), where the parameters are given as $q_{1}=6275.9$, $q_{2}=1.0630$, $q_{3}=0.7161$, and $q_{4}=1942.8$.

(16) $$F\left(L\right)=q_{1}+\frac{q_{1}-q_{4}}{1+{\left(\frac{L}{q_{3}}\right)}^{q_{2}}}$$

**Figure 13:**
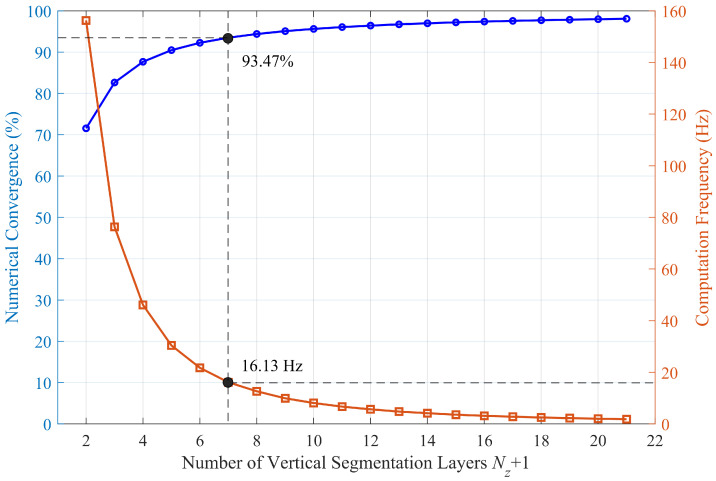
The numerical convergence and computation frequency of the magnetic force estimation framework varying with number of segmentation layers.

Based on this fitting, the asymptotic limit of the force can be estimated, which is 6275.9 N. To present the analysis more intuitively, the estimated force is normalized by the asymptotic limit to derive the numerical convergence, and the reciprocal of the computation time is defined as the computation frequency. In this way, an accuracy–efficiency trade-off curve is obtained, as shown in Figure 13. It can be observed that when the number of slices is small, the computation is efficient, but the convergence is insufficient to meet practical requirements. Conversely, when the number of slices increases, the improvement in convergence becomes marginal, while the computational cost increases significantly, which degrades the real-time performance of the framework. Considering the trade-off between accuracy and efficiency, the number of slices was finally set to 7. At this setting, the estimated force reaches 93.47% of the asymptotic limit while maintaining a computation frequency exceeding 16 Hz, achieving a balanced compromise suitable for real-time applications. The results of the force estimation and the average computation time are compared in Section 3.

**Figure 14:**
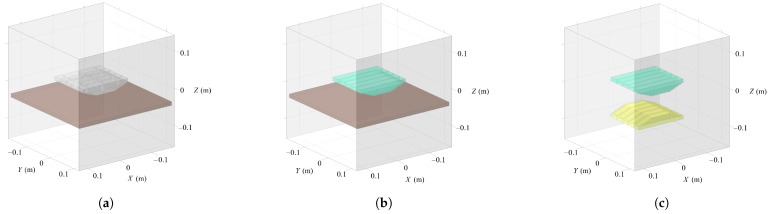
Configuration of three different simulation scenes in COMSOL Multiphysics. (**a**) The original Halbach PMA with the steel plate. (**b**) The equivalent Halbach PMA with the steel plate. (**c**) The equivalent Halbach PMA with its image array.

**Figure 15:**
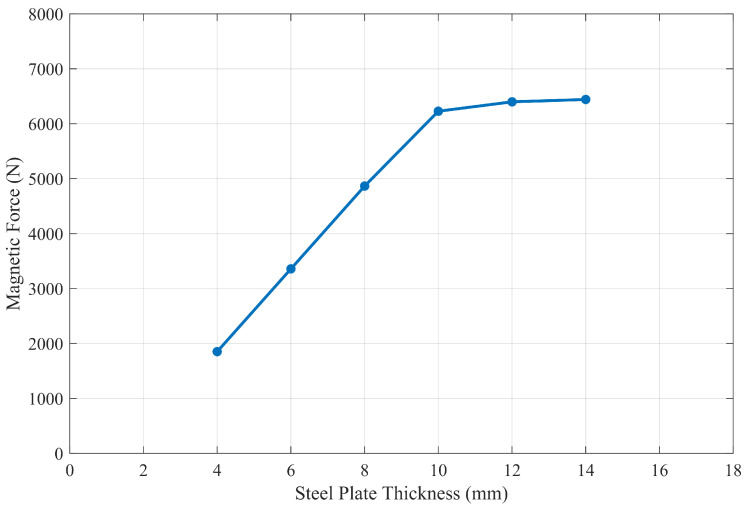
Effect of steel plate thickness on magnetic adhesion force.

Considering the effect of the simplification and equivalent method for the magnets, three different scenes were simulated in COMSOL Multiphysics, including the original Halbach PMA with the large steel plate, the equivalent Halbach PMA with the large steel plate, and the equivalent Halbach PMA with its image array. The visualization of the scenes are illustrated in Figure 14. The simulation results are presented in Section 3, where the three scenes are denoted as “COMSOL (Original)”, “COMSOL (Equivalent)”, and “COMSOL (MoI)”, respectively. All magnetic field computations were conducted within a cubic air domain with a side length of 0.3 m as Figure 14 depicts. To be consistent with the physical experimental setup, the material of all magnets in simulation is set to NdFeB BMN-52 in the COMSOL material library, with a remanent magnetization specification of 1.45 T, while the large steel plate is made of Q235 structural steel (approximately equivalent to ASTM A36/EN S235). The B–H curve data of the Q235 steel used in the simulation are provided in the Appendix A Table A1. Since the ideal MoI model requires a sufficiently thick steel plate to avoid magnetic saturation effects, a parametric study was conducted by simulating the magnetic adhesion force corresponding to different plate thicknesses to determine the critical condition. In the simulations, the plate thickness was varied from 4 mm to 14 mm with an increment of 2 mm, and the resulting variation in magnetic adhesion force with respect to the plate thickness is shown in Figure 15. It can be observed that when the plate thickness increases to 10 mm, the system effectively operates outside the magnetic saturation region, and the estimation error caused by flux leakage becomes negligible. Therefore, in the practical magnetic adhesion force measurement experiment, selecting a steel plate with a thickness of 10 mm is considered acceptable and also consistent with commonly used thicknesses in industrial steel structures.

**Figure 16:**
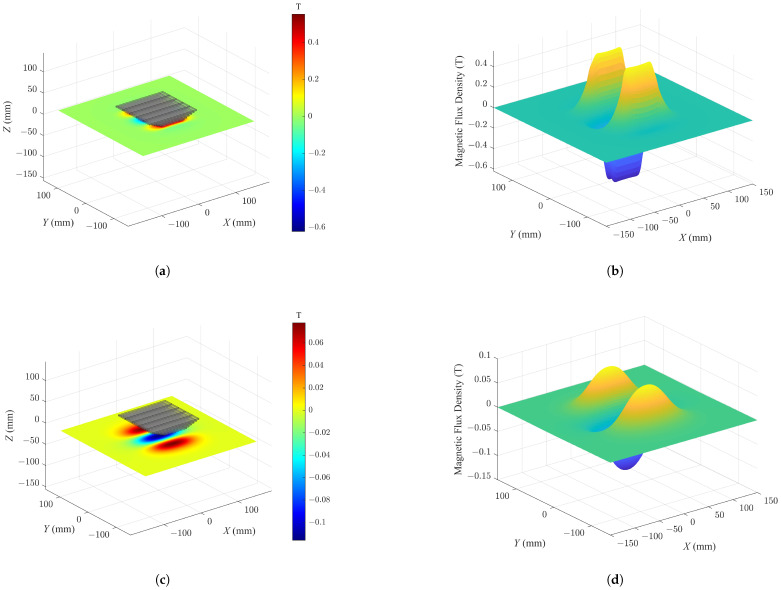
Simulated magnetic flux density distribution on the steel plate surface generated by the complex-shaped PMA. (**a**) MagTetris visualization when air gap is 2 mm; (**b**) 3D surface plot when air gap is 2 mm; (**c**) MagTetris visualization when air gap is 29 mm; (**d**) 3D surface plot when air gap is 29 mm.

In addition to the thickness of the steel plate, the effect of the dimensions in the length and width directions was also taken into consideration. As shown in Figure 16, the magnetic flux density distribution generated by the PMA on the steel plate surface was simulated and visualized through MagTetris. As shown in the figure, regardless of the air-gap distance, all regions exhibiting nonzero magnetic flux density are fully contained within the in-plane dimensions of the simulated steel plate. The magnetic force can be interpreted as arising from the spatial variation in the magnetic field, i.e., the gradient of magnetic energy. As shown in the figure, only the region with non-negligible magnetic flux density contributes to the force. In contrast, regions where the magnetic flux density approaches zero provide no contribution, since the corresponding field gradient vanishes. Based on this observation, it can be inferred that the selected in-plane dimensions of the steel plate are sufficiently large such that boundary effects become negligible, and the system can be reasonably approximated as satisfying the ideal conditions required for the applicability of the MoI model.

**Figure 17:**
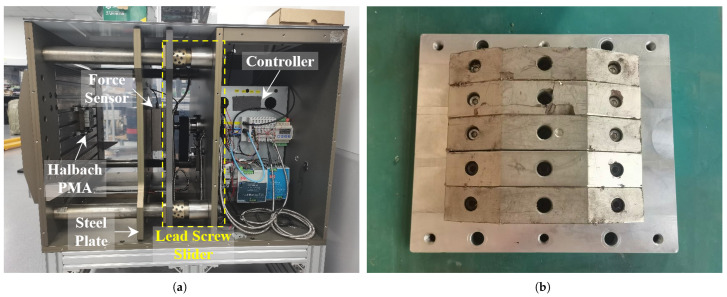
(**a**) Magnetic force measurement experimental platform. (**b**) The full-scale manufactured and assembled Halbach PMA.

To verify the accuracy of the proposed method, magnetic adhesion force measurement experiments were conducted using full-scale physical magnets. The experimental platform is shown in Figure 17a, which was used to measure the magnetic adhesion force between a linear Halbach PMA and a large steel plate. The Halbach PMA was fixed on a vertically oriented wall, while the steel plate was mounted on a horizontal lead-screw slider module with one side facing the PMA and the other connected to the slider via a force sensor. In this configuration, the controller drives the lead-screw module to adjust the air-gap distance between the Halbach array and the steel plate, and the magnetic adhesion force acting on the steel plate is recorded by the force sensor. A full-scale manufactured Halbach PMA according to the model studied previously, as Figure 17b shows, was mounted in the platform for the force measurement. The magnets and the steel plate are made of the materials described above in the COMSOL simulation setup. The estimation errors of the magnetic adhesion force obtained by the aforementioned methods were evaluated using the following error rate:

(17) $$\varepsilon =\left|\frac{F_{m}-F_{e}}{F_{m}}\right|$$

where $F_{m}$ denotes the magnitude of the measured force, and $F_{e}$ represents the magnitude of the estimated force. The comparison of the error rates of different estimation methods is presented in Section 3.

**Figure 18:**
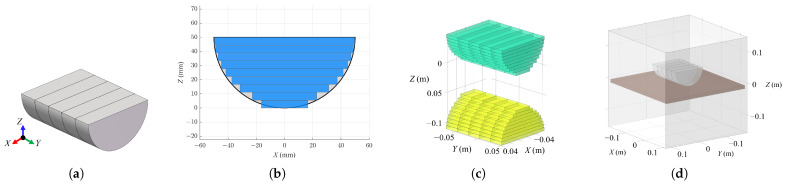
(**a**) CAD model of the semi-cylindrical Halbach PMA (**b**) The gray region denotes the semicircular cross-section and the blue boxes represent the slice set output by the 2D conservative slicing. (**c**) The source and image array constructed from the semi-cylindrical Halbach PMA. (**d**) COMSOL simulation configuration of the semi-cylindrical PMA.

To further verify the generality of the proposed slicing and approximation strategy, an additional PMA configuration composed of semi-cylindrical permanent magnets was introduced for simulation validation. The PMA adopts the same linear Halbach array configuration consisting of five PMs. The spatial arrangement, magnetization permutation, and thickness in the y-direction are identical to those of the previously described complex-shaped PMA; the only difference lies in the geometry of the magnets. Each magnet has a semicircular cross-section with a radius of 50 mm and is approximated using a nine-layer slicing scheme, as illustrated in Figure 18. The curved profile of the semicircle is discretized into polygon first to be. The COMSOL simulation settings are consistent with those described earlier. In this case, only a single scenario—namely, the combination of the original magnets and the steel plate—is considered. The comparison between the simulation results and the predictions of the proposed framework is presented in Section 3.

## 3. Results

**Figure 19:**
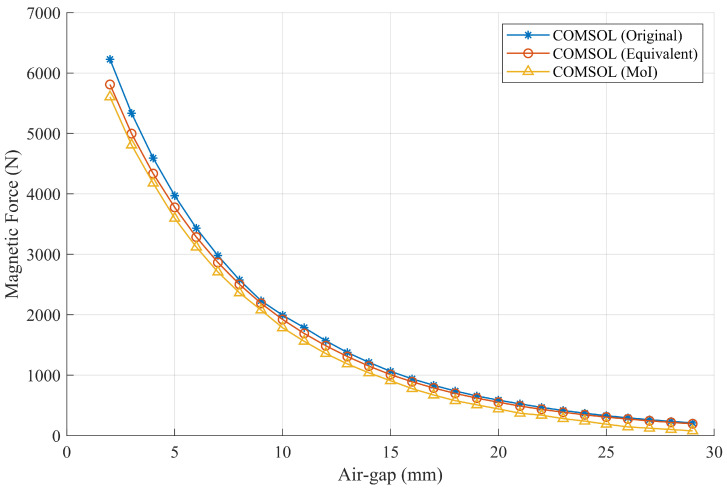
Comparison of magnetic force estimation results in COMSOL using different models.

The force computation results of the three simulation scenes in COMSOL Multiphysics are illustrated in Figure 19. Due to the high symmetry of the studied PMA and its parallel alignment with the wall surface, the magnetic adhesion force reported in the following results corresponds to the *Z*-direction component of the resultant magnetic force and is not explicitly indicated in the figures. The resultant force components in the other two directions are negligible and therefore not discussed further. From the overall trends and the close agreement among the curves, it can be observed that the magnet segmentation approximation and the magnetostatic MoI introduce negligible error in the estimation of the magnetic adhesion force.

The average computation time of different methods is listed in Table 1. Each value represents the average time obtained from 50 force calculations. As the computation timing results reveal, compared with the FEM and field-based method, the proposed framework shows a significant advance in terms of efficiency. Although the MagTetris framework already exhibits a noticeable improvement in computational efficiency compared with the FEM, it still requires more computation time because the magnetic adhesion force is obtained indirectly through the superposition of discretized magnetic flux density integration. In contrast, the proposed method directly computes the magnetic force through closed form equations, which further improves computational efficiency. Therefore, the proposed method is capable of meeting the real-time engineering requirements of magnetic adhesion force estimation in wall-climbing robots.

**Figure 20:**
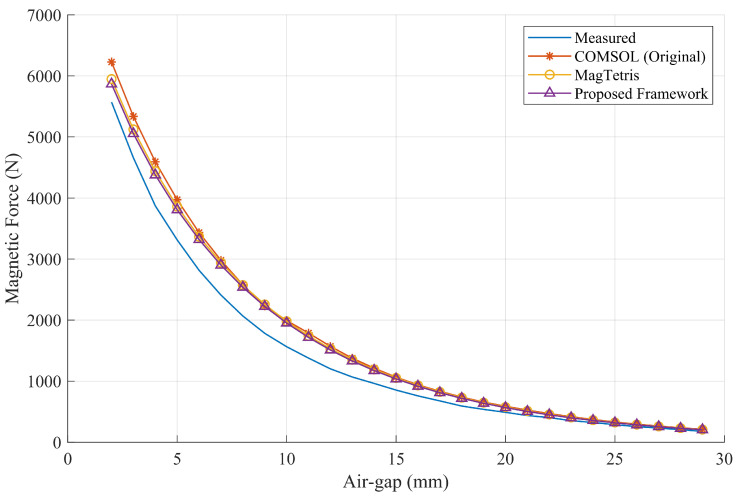
Comparison of magnetic force estimation results of the complex-shaped PMA.

**Figure 21:**
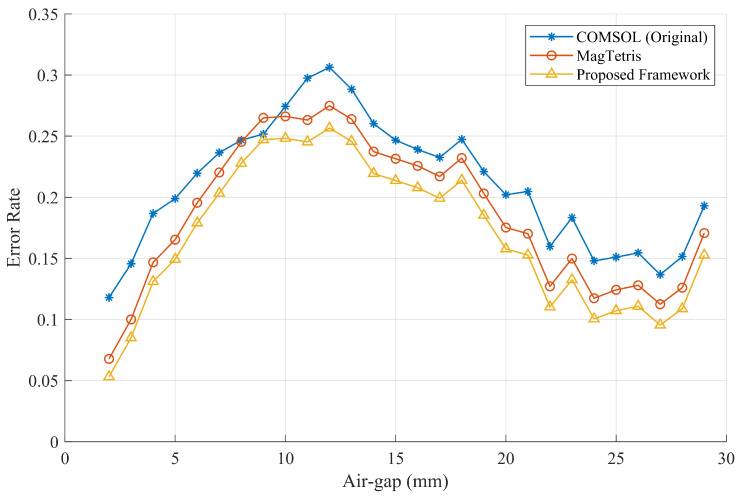
Comparison of magnetic force estimation error with respect to the measured data of the complex-shaped PMA.

**Figure 22:**
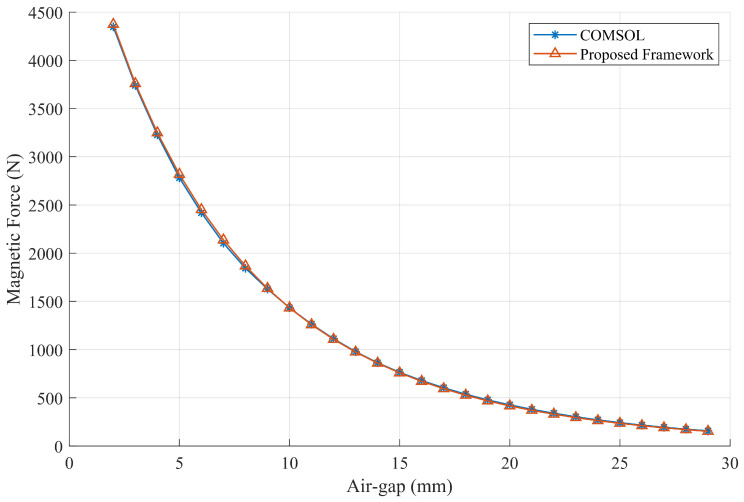
Comparison of magnetic force estimation results of the half-cylinder PMA.

The recorded magnetic forces that change with the air gap, along with the force estimation results of different mathods, are presented in Figure 20. It can be observed from the data that the magnetic adhesion force estimated by the proposed framework is highly consistent with the results obtained using the FEM method and the MagTetris framework. Furthermore, as shown by the error rates in Figure 21, certain deviations between the estimated and measured forces can be observed due to factors such as manufacturing and assembly errors, variations in magnetization, and material anisotropy. Nevertheless, since the results obtained by the different estimation methods are highly similar, the estimation accuracy of the proposed method can still be effectively validated. Moreover, as illustrated in Figure 22, for the semi-cylindrical magnet, the proposed framework is also capable of accurately estimating the magnetic adhesion force, demonstrating its generalizability to a certain extent.

## 4. Conclusions

This paper presents a real-time magnetic adhesion force estimation framework for wall-climbing robots with Halbach permanent magnet arrays and adjustable air gaps. By integrating the magnetostatic MoI, 3D analytical formulations, and cuboid-based segmentation, the proposed approach enables efficient and accurate force evaluation.

The MoI-based model replaces the ferromagnetic wall with equivalent image magnets, transforming the problem into magnet–magnet interactions and avoiding computationally intensive magnetization calculations. Meanwhile, the segmentation strategy extends the framework to complex magnet geometries, including Halbach arrays and magnets with polygonal cross-sections.

Both numerical and experimental results demonstrate that the proposed method achieves accuracy comparable to FEM and existing analytical approaches while significantly improving computational efficiency.

It should be noted that the current study is limited to offline validation of the proposed framework, and its deployment within a real-time robotic control system has not yet been realized. Therefore, further investigations are necessary to comprehensively assess its integration feasibility and practical adaptability in real-world control scenarios.

Overall, the framework provides a practical solution for real-time magnetic adhesion force estimation, with strong applicability to complex magnet configurations and ferromagnetic surfaces. Future work will focus on extending the proposed framework to more complex and realistic conditions, including non-planar surfaces, discontinuous or segmented wall structures, and other challenging boundary conditions. In addition, further efforts will be devoted to accommodating magnets with nonuniform thickness and irregular geometries, thereby enhancing the model’s capability to handle highly complex and practical configurations. Meanwhile, the integration of the framework into real-time robotic control systems will be investigated to evaluate its performance and adaptability in practical applications.

## Figures and Tables

**Table 1:** Average computation time comparison for different force estimation methods.

Method	Model	Average Computation Time (s)
COMSOL (MoI)	Numerical	15.7
MagTetris	Field-based analytical	5.41
Proposed framework	Force-based analytical	0.0621

## Data Availability

The data presented in this study are available upon reasonable request from the corresponding author.

## Nomenclature

List of symbols and abbreviations used in this manuscript

Symbol	Description	Unit
P	Polygonal cross-section of the magnet	–
$\left(x_{k},z_{k}\right)$	Vertex coordinates of the polygon	m
*N*	Total number of cuboid elements after segmentation	–
$N_{z}$	Number of slices along the *z*-direction	–
$z_{i}$	Center coordinate of the *i*-th slice	m
Δz	Thickness of each slice	m
$x_{L},x_{R}$	Left and right boundaries of a slice interval	m
$x_{i}$	Center position of a slice	m
$l_{i}$	Width of a slice	m
α,β,γ	Relative displacement between magnets along *x*, *y*, *z*	m
A,B,C	Half-dimensions of one magnet along *x*, *y*, *z*	m
a,b,c	Half-dimensions of the other magnet along *x*, *y*, *z*	m
*r*	Distance between two interacting points	m
F	Total magnetic adhesion force vector	N
$\Delta F_{i}$	Force contribution of the *i*-th cuboid element	N
$\Delta F_{ij}$	Force between source and image magnets	N
$M,M^{\prime }$	Magnetization vectors of interacting magnets	A/m
$\mu_{0}$	Vacuum permeability	H/m
ϕ	Analytical kernel function for magnetic force computation	–
$F_{m}$	Measured magnetic force	N
$F_{e}$	Estimated magnetic force	N
ε	Relative error of force estimation	–
$q_{1},q_{2},q_{3},q_{4}$	Parameters of the logistic fitting function	–
F(L)	Estimated force as a function of slice number	N
$U_{ij},V_{kl},W_{pq}$	Auxiliary variables in analytical force computation	m
Abbreviation	Full Term	
PM	Permanent Magnet	
PMA	Permanent Magnet Array	
FEM	Finite Element Method	
MoI	Method of Images	
MEC	Magnetic Equivalent Circuit	

## Appendix A

**Table A1:** Discrete B–H curve data of Q235 steel used for interpolation in COMSOL Multiphysics.

Magnetic Flux Density *B* (T)	Magnetic Field Intensity *H* (A/m)
0.00	0
0.20	100
0.50	200
1.00	500
1.40	1000
1.60	2000
1.70	4000
1.75	8000
1.78	16,000
1.80	30,000
